# Investigation of salicylate hepatic responses in comparison with chemical analogues of the drug

**DOI:** 10.1016/j.bbadis.2016.04.015

**Published:** 2016-08

**Authors:** Amy R. Cameron, Lisa Logie, Kashyap Patel, Sandra Bacon, Calum Forteath, Jean Harthill, Adam Roberts, Calum Sutherland, Derek Stewart, Benoit Viollet, Kei Sakamoto, Gordon McDougall, Marc Foretz, Graham Rena

**Affiliations:** a Cardiovascular and Diabetes Medicine, Ninewells Hospital and Medical School, University of Dundee, Dundee, Scotland DD1 9SY, United Kingdom; b MRC Protein Phosphorylation and Ubiquitylation Unit, College of Life Sciences, University of Dundee, Dundee, Scotland DD1 5EH, United Kingdom; c James Hutton Institute, Invergowrie, Dundee, Scotland DD2 5DA, United Kingdom; d School of Life Sciences, Heriot-Watt University, Edinburgh, Scotland EH14 4AS, United Kingdom; e INSERM U1016, Institut Cochin, Paris 75014, France; f CNRS UMR8104, Paris 75014, France; g Université Paris Descartes, Sorbonne Paris Cité, Paris 75014, France; h Nestlé Institute of Health Sciences SA, EPFL Innovation Park, Bâtiment G, 1015 Lausanne, Switzerland

**Keywords:** Salicylate, AMPK, mTOR signalling, NF-κB signalling, Gluconeogenesis

## Abstract

Anti-hyperglycaemic effects of the hydroxybenzoic acid salicylate might stem from effects of the drug on mitochondrial uncoupling, activation of AMP-activated protein kinase, and inhibition of NF-κB signalling. Here, we have gauged the contribution of these effects to control of hepatocyte glucose production, comparing salicylate with inactive hydroxybenzoic acid analogues of the drug. In rat H4IIE hepatoma cells, salicylate was the only drug tested that activated AMPK. Salicylate also reduced mTOR signalling, but this property was observed widely among the analogues. In a sub-panel of analogues, salicylate alone reduced promoter activity of the key gluconeogenic enzyme glucose 6-phosphatase and suppressed basal glucose production in mouse primary hepatocytes. Both salicylate and 2,6 dihydroxybenzoic acid suppressed TNFα-induced IκB degradation, and in genetic knockout experiments, we found that the effect of salicylate on IκB degradation was AMPK-independent. Previous data also identified AMPK-independent regulation of glucose but we found that direct inhibition of neither NF-κB nor mTOR signalling suppressed glucose production, suggesting that other factors besides these cell signalling pathways may need to be considered to account for this response to salicylate. We found, for example, that H4IIE cells were exquisitely sensitive to uncoupling with modest doses of salicylate, which occurred on a similar time course to another anti-hyperglycaemic uncoupling agent 2,4-dinitrophenol, while there was no discernible effect at all of two salicylate analogues which are not anti-hyperglycaemic. This finding supports much earlier literature suggesting that salicylates exert anti-hyperglycaemic effects at least in part through uncoupling.

## Highlights

• Pharmacology and knockout approaches were used to study salicylate in hepatocytes.
• AMPK is not required for salicylate to inhibit NF-κB signalling.
• Inhibition of neither NF-κB nor mTOR can suppress hepatocyte glucose production.
• Salicylate acts as an uncoupler at a physiologically relevant dose.
• Uncoupling properties are likely to contribute to anti-hyperglycaemic drug action.

## 1 Introduction

Our recent work has investigated the mechanism of action of the biguanide metformin [1], [2], [3] using chemical analogues of the drug. We have become interested in widening this approach to study other anti-hyperglycaemic agents, particularly those that share responses with biguanides. For the current study, we have focused on the anti-hyperglycaemic effects of the hydroxybenzoic acid (HBA) salicylate (SA), which require much higher concentrations than are required to inhibit prostaglandin production, suggesting that other mechanisms contribute to their antidiabetic effects [4]. AMP-activated protein kinase (AMPK) is an important focus of research, due to the discovery that salicylates and other anti-hyperglycaemic agents including biguanides and glitazones share in common an ability to activate AMPK [5], [6], [7], [8], [9]. This enzyme, which is activated by energy stress (for example, elevated [AMP]), acts as a cellular energy checkpoint, suppressing ATP-consuming processes and promoting ATP-generation [10], [11]. Before recent studies on salicylate and AMPK [12], work on salicylate and related drugs in the 1950s suggested that anti-hyperglycaemic efficacy might be related to uncoupling effects [13], [14], [15], [16]. One study in the 1970s found that SA suppressed hepatic gluconeogenesis [17], but in recent years, inflammatory signalling mechanisms, particularly inhibition of TNF-α-induced NF-κB signalling [9], [18], [19], [20], have become more prominent. Other workers have found that TNF-α-dependent activation of NF-κB suppresses gluconeogenesis [21], suggesting that effects of SA on the mitochondria and NF-κB could even oppose each other at the level of gluconeogenesis.

It is very difficult to distinguish the relative contribution of these responses by genetic modification, not least because the uncoupling effect is unlikely to require interaction with any specific gene product. Genetic knockout of IKKβ [20] improves glucose tolerance akin to treatment with SA; however, it has not yet been possible to demonstrate genetic blockade of anti-hyperglycaemic effects of SA. For example, SA significantly improves glucose tolerance in AMPK-knockout mice [12], and other gene-targeting studies with metformin indicate that repression of hepatic gluconeogenesis with this agent can proceed in an AMPK-independent manner [2], [22]. In the current study, exploitation of the chemical analogue approach, involving comparison of SA with other HBAs, has afforded an excellent opportunity to investigate which of these cell responses correspond best with known anti-hyperglycaemic responses to the drugs.

## 2 Materials and methods

### 2.1 Materials

The compounds used in this study were dissolved directly in DMEM and the pH corrected to pH7.4. The phospho-acetyl-CoA carboxylase (ACC) Ser 79 antibody was from the Division of Signal Transduction Therapy at the University of Dundee. The total ACC, total AMPKα, phospho-AMPKα Thr 172, total S6, phospho-S6 Ser 240/244, phospho-p70S6K Thr 389, total IκB, pNF-κB, total IKKα, and total IKKβ antibodies for immunoblotting were from Cell Signaling Technology. Actin antibody was from Merck. Antibodies used in the AMPK activity assays were a generous gift from Prof D. Grahame Hardie at the University of Dundee. Chemical structures were drawn using ChemSketch. BI605906 was a generous gift from Prof Sir Philip Cohen (MRC Protein Phosphorylation and Ubiquitylation Unit, Dundee).

### 2.2 Cell culture and lysis for immunoblotting

H4IIE cells were maintained essentially as described previously [1], [23], [24], [25] grown in DMEM plus 5% Fetal calf serum (Seralab) and used for no more than 30 passages. Briefly, fresh medium was added the evening before an experiment and cells were lysed on the fifth or sixth day after seeding. Two hours prior to stimulation, cells were placed in DMEM without serum. For lysis, cells were scraped into ice-cold buffer A: (50 mM Tris acetate pH7.5, 1% (*w*/*v*) Triton X100, 1 mM EDTA, 1 mM EGTA, 0.27 M sucrose, 50 mM NaF, 1 mM sodium orthovanadate, 10 mM β glycerophosphate, 5 mM sodium pyrophosphate, 1 mM benzamidine, 0.2 mM phenylmethylsulfonyl fluoride (PMSF), and 0.1% (*v*/v) β-mercaptoethanol) and then prepared for SDS-PAGE as described previously [26], [27]. The protein concentration was measured using Bradford reagent (Pierce). HT-29 cells were a generous gift from Prof. Inke Nathke (Dundee). They were grown similarly to H4IIE cells except that they were cultured in 4.5 g/l glucose-containing DMEM supplemented with 10% serum (PAA) and non-essential amino acids (Sigma). Extraction of primary hepatocytes was carried out essentially as described previously [1], [22]. Immunoblot densitometry for each antibody was performed with Image Studio Lite version 5.2 (LI-COR).

### 2.3 Preparation of cell extracts, immunoprecipitation and assay of AMPK

This was carried out essentially as described previously [1]. Briefly, cells were washed twice in ice-cold PBS then harvested in ice-cold lysis buffer (50 mM Tris–HCl, pH 7.4, 50 mM sodium fluoride, 5 mM sodium pyrophosphate, 1 mM EDTA, 1 mM EGTA, 150 mM sodium chloride, 1 mM dithiothreitol (DTT), 0.1 mM benzamidine, 0.1 mM PMSF, 1% Triton X-100, and 5 μg/ml soybean trypsin inhibitor). Lysates were cleared of debris by centrifugation at 13,000*g* for 15 min at 4 °C, and the protein concentration measured as in the previous section. AMPK assay was carried out essentially as described previously [1]. Briefly, cell extracts were incubated overnight with protein G sepharose conjugated to both anti-AMPKα1 and AMPKα2 antibodies [28]. Immunoprecipitates were pelleted and rinsed twice with 1 ml ice-cold buffer (as above but with 0.5 M NaCl) and once with ice-cold HEPES buffer (50 mM HEPES pH 7.4, 0.03% Brij-35, and 1 mM DTT). AMPK activity was assayed at 30 °C, in the presence of 0.1 μCi of [γ-^32^P]ATP, 0.33 mM cold ATP, 8.3 mM MgCl_2_, 0.33 mM AMP, and 0.33 mM SAMS peptide. Kinase activity is expressed as the amount of AMPK catalyzing the phosphate incorporation of 1 nmol substrate in 1 min per mg of protein. Each bar of a graph consists of data from at least six separate immunoprecipitations, each from a separate dish of cells. All animal care protocols and procedures were performed in accordance with current regulations.

### 2.4 Generation of LLHG glucose 6-phosphatase (G6Pase) promoter reporter cell line

The human G6Pase promoter was cloned using genomic DNA extracted from HepG2 cells. Briefly, the promoter region stretching from − 2785 bp to + 85 bp, relative to the transcriptional start site, was amplified using the following primers: Human G6Pase fwd 5′–GTCGACCCTTTGAGAATCCACGGTGTC–3′ and Human G6Pase rev 5′–AAGCTTAGGTGCCAAGGAAATGAGG − 3′. The G6Pase promoter was first sub-cloned into TOPO before further cloning into pGL4.17[luc2/neo] (Promega) using KpnI and XhoI and confirmed correct by DNA sequencing. H4IIE cells were transfected with the pGL4-Human G6Pase construct using the calcium phosphate transfection methodology [29]. Cells that had stably integrated the pGL4-human G6Pase-luciferase DNA were selected by treating with 500 μg/ml G418 for 1 week. Surviving colonies were expanded, in the presence of G418, until they could be screened for luciferase expression.

Transfected cells were screened for hormonal regulation of the G6Pase promoter. Conditions of fasting and feeding were mimicked by the addition of dexamethasone (500 nM), 8-CPT-cAMP (100 μM), and insulin (10 nM), respectively, and cells which showed significant stimulation and repression of luciferase expression were then further examined to verify that key components of the insulin signalling pathway [30], [31], [32], including PI-3 kinase, PKB, and GSK3 were responsive (data not shown). The cell line LLHG was selected for future use.

### 2.5 Luciferase reporter assay

LLHG cells were seeded onto 12-well plates and left overnight. Cells were washed once in serum-free DMEM before serum starving for 6 h and subsequent overnight treatment as indicated in figure legends. For lysis, cells were washed once in PBS before addition of 1 × Cell Culture Lysis Buffer (Promega). Wells were scraped and lysates centrifuged for 2 min, 4 °C, 13,000 rpm. For luciferase assay, 10 μl lysate was added to a 96-well, white walled, clear bottom plate and 100 μl luciferase assay reagent (Promega) added before mixing briefly and quantifying luminescence. Values were normalized to lysate protein concentration by Bradford assay. Each bar of a graph consists of data from at least six separate measurements, each from a separate dish of cells.

### 2.6 Glucose assay

Treatment of cells for hepatocyte glucose production was carried out essentially as described previously, using primary mouse hepatocytes with modifications [1], [22]. Glucose production was determined after a 12-h incubation period in glucose-free DMEM with or without HBAs, other drugs or 2 mM metformin. At the end of the incubation period, medium was collected and glucose concentration determined by fluorescence measurement in the Amplex Red glucose assay (Invitrogen) for experiments including hydroxybenzoic acid or GAGO assay (Sigma) for other experiments. Each bar of a graph consists of data from at least three separate measurements, each from a separate dish of cells.

### 2.7 Measurement of whole-cell oxygen consumption rate

Using the Seahorse XF96 (Seahorse Bioscience), oxygen consumption rate (OCR) was measured essentially as described previously [12]. Briefly, H4IIE cells were plated at a density of 3 × 10 [4] cells/well in 80 μl serum-containing medium and incubated overnight. For the assay, cells were washed twice and incubated for 2 h in 200 μl serum-free medium (Phenol red free DMEM [A14430 Gibco], 25 mM HEPES, 5.5 mM glucose, 2 mM L-Glut, and 2.5 mM pyruvate). 180 μl fresh media was added and the plate was degassed at 37 °C for a further 1 h. OCR was continuously measured for a period of 50 min. 20 μl of 10X stock drugs made up in the same SF media were added after the first baseline reading.

### 2.8 Data analysis

Data are expressed as mean ± SEM. Statistical analyses were performed using one-way ANOVA with Dunnett's or Tukey's post hoc testing using GraphPad Prism 6 statistical software.

## 3 Results

### 3.1 Comparison of SA and related compounds on AMPK and mTOR signalling

**Fig. 1:**
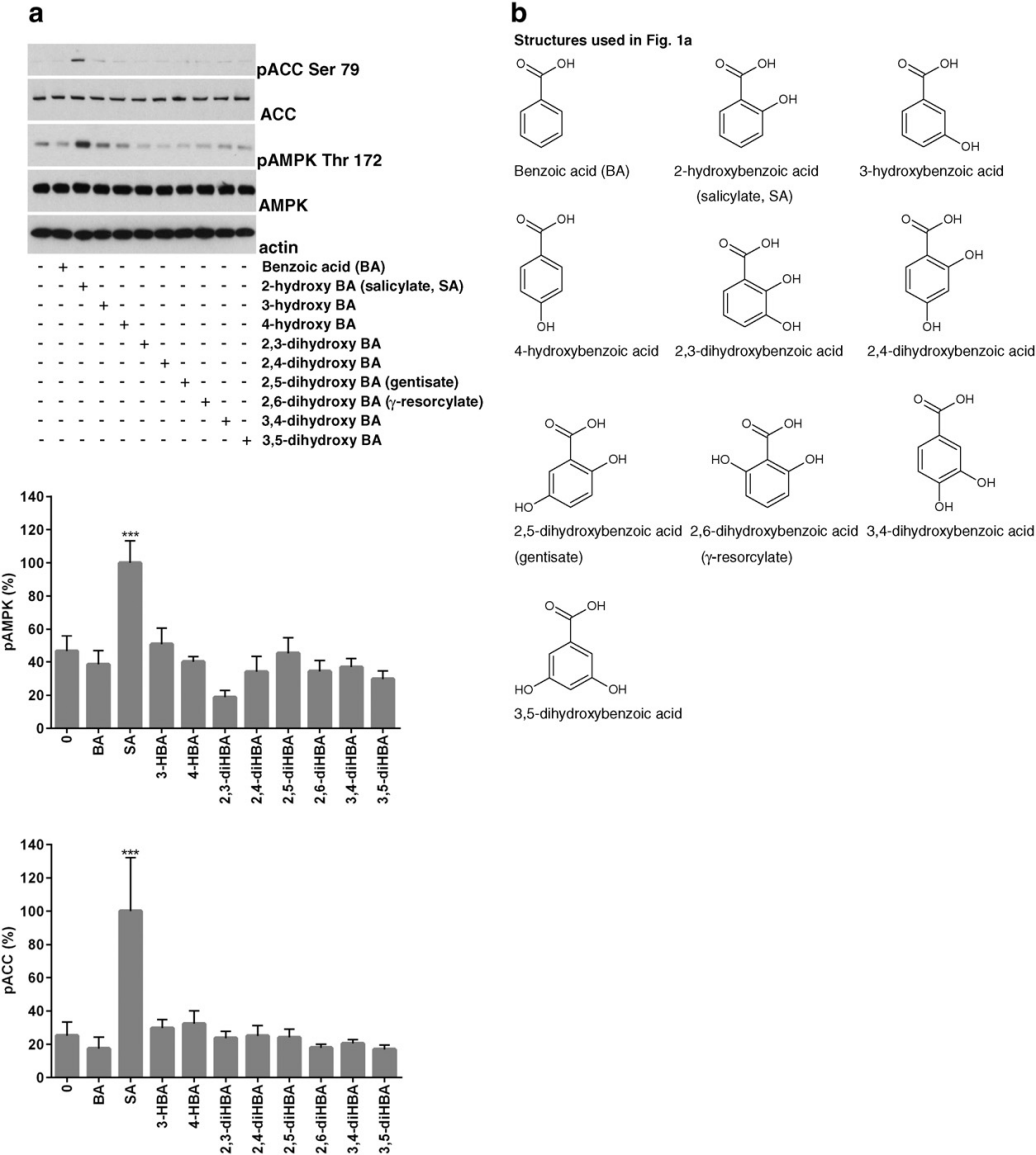

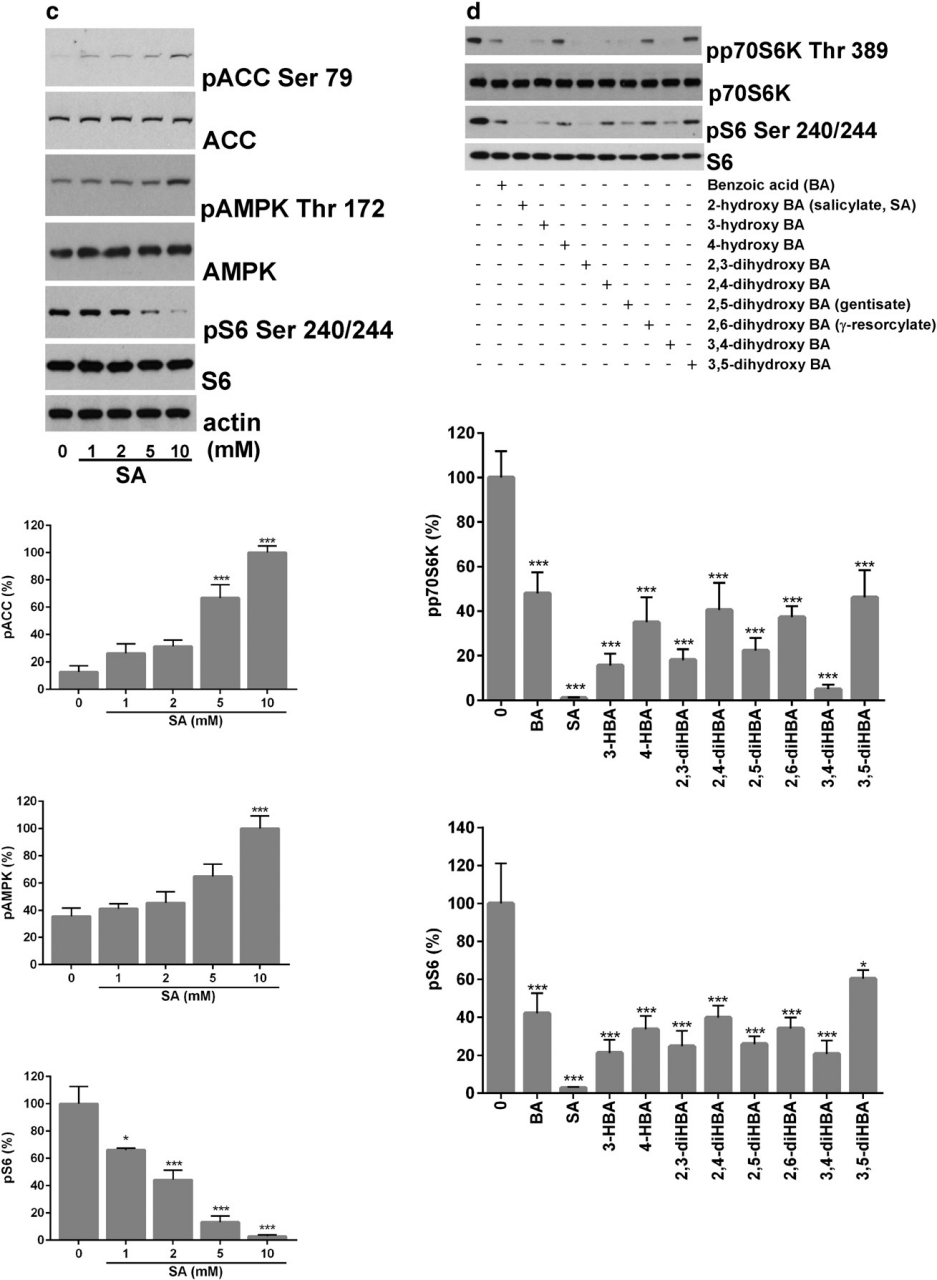
*Comparison of effects of SA and analogues on AMPK signalling*. (*a*,*b*) H4IIE cells were incubated in serum-free medium for 2 h, prior to stimulation for 3 h with or without the agents (10 mM) shown. Densitometry of blots from each experiment was carried out as described in the Materials and methods. Treatments significantly different from untreated cells are shown; ^⁎⁎⁎^p < .001, n = 5. Cells were then lysed and prepared for immunoblotting as described in the methods. Two acetyl-CoA carboxylase (ACC) antibodies were used, one which detects total ACC1/2 (ACC) and one which detects phosphorylated ACC1/2 (pACC Ser 79). A third and fourth antibody detects AMPK whether or not it is phosphorylated. A fifth antibody is to the housekeeping protein actin. (*c*) H4IIE cells were incubated in serum-free medium and then stimulated as in (*a*) but with a dose–response of SA as shown. Besides the antibodies used earlier, two S6 antibodies were used, one which detects total S6 protein (S6) and another one which detects phosphorylated S6 protein (pS6 Ser 240/244). Cells were then lysed and prepared for immunoblotting with antibodies as already described in the methods; ^⁎^p < .05, n = 3–4. (*d*) Lysates treated as in (*a*) were probed with the S6 antibodies just described. Two further antibodies, to detect p70S6K regardless of phosphorylation (p70S6K) or when phosphorylated on residue Thr389 (pp70S6K Thr389), were also used, n = 4.

In a panel of drugs related to SA, we found that SA alone induced phosphorylation of AMPK and its substrate ACC strongly, while other compounds elicited less robust responses, if any (Fig. 1a). The structures used are shown in Fig. 1b. In dose–response experiments, SA also reduced phosphorylation of S6 (Fig. 1c), which is a reporter of mTOR signalling that is regulated by metformin [1], [33], [34] and thiazolidinediones [1]. Next, we investigated further the mTOR signalling pathway responses to the various SA analogues (Fig. 1d). AMPK-dependent and independent mechanisms have been suggested to account for effects of metformin on mTOR signalling [33], [35]. Possibly consistent with more than one mechanism, we found that mTOR pathway suppression is a common property of almost all HBAs (Fig. 1d).

### 3.2 Investigation of NF-κB, AMPK, and gluconeogenic gene expression responses to hydroxybenzoic acids and their analogues

**Fig. 2:**
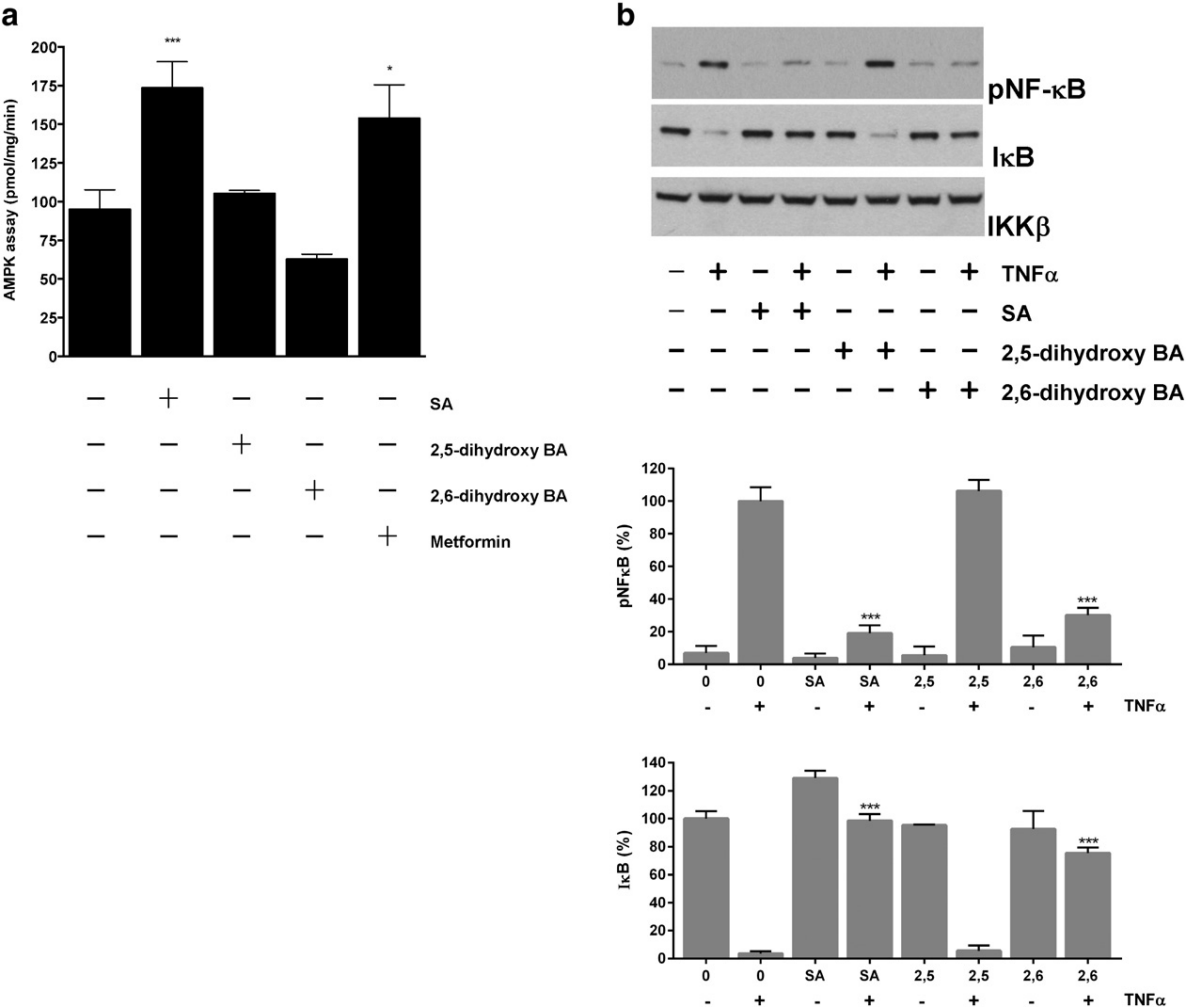
*Effect of SA and analogues on AMPK activation and NF-κB signalling.* (*a*) H4IIE cells were grown in serum-free medium for 2 h, followed by stimulation with the agents shown (10 mM) for 3 h followed by AMPK assay as described in Materials and methods. Treatments significantly different from untreated cells are shown; ^⁎⁎⁎^p < .001, ^⁎^p < .05 with respect to untreated cells. (*b*) HT-29 cells were serum starved overnight in 0.25% serum, prior to treatment for 1 h without or with 30 mM each of the analogues shown. The effect of each agent on IκB levels following 10 ng/ml TNF-α treatment (final 15 min) was assessed by immunoblotting. Another antibody detected total IKKβ. Densitometry of blots was carried out as described in Materials and methods. Co-incubations of drug with TNF-α significantly different from TNF treatment alone are shown. For both antibodies, TNF-α plus salicylate n = 7, TNF-α plus 2,6-DHBA n = 4.

We continued to study more intensively SA analogues with better characterised pharmacology. These were 2,5-DHBA (also known as gentisate), 2,6-DHBA (also known as γ-resorcylate), both of which have been reported to have anti-inflammatory properties by some but not all investigators [36], [37], [38], [39], but neither of which exhibit short-term anti-hyperglycaemic properties *in vivo* [14], [36], [40]. In H4IIE cells, we used AMPK assays to confirm our earlier immunoblotting data that SA is the only AMPK activator in this focussed panel, with no other agent having significant effects on AMPK activity compared with untreated cells (Fig. 2a).

To investigate anti-inflammatory signalling effects of the panel, we studied the effects of each drug on blockade of TNFα-dependent degradation of IκB in HT-29 cells. We chose this cell line because it has been used previously to study salicylate effects on inflammatory signalling [18]. In the panel, 2,6-DHBA was capable of repressing IκB degradation in a similar manner to SA, whereas 2,5-DHBA was unable to protect IκB from degradation (Fig. 2b).

### 3.3 Effect of the selected agents on hepatic signalling

**Fig. 3:**
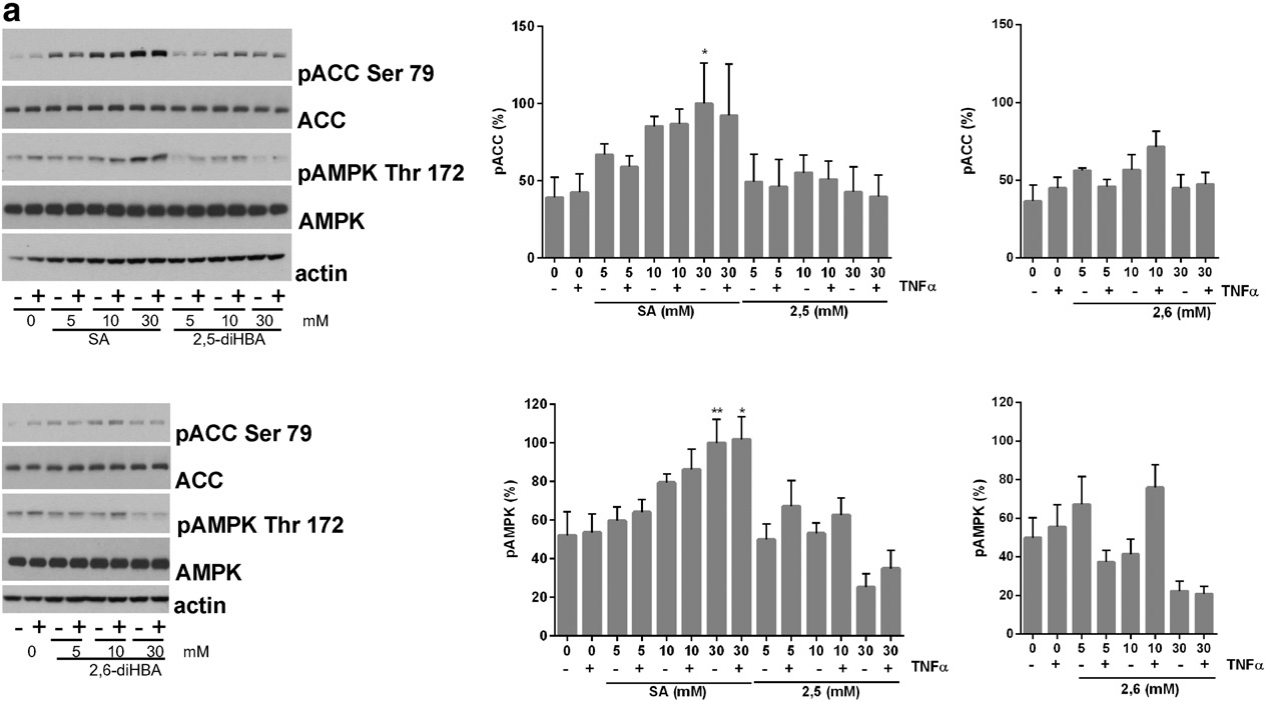

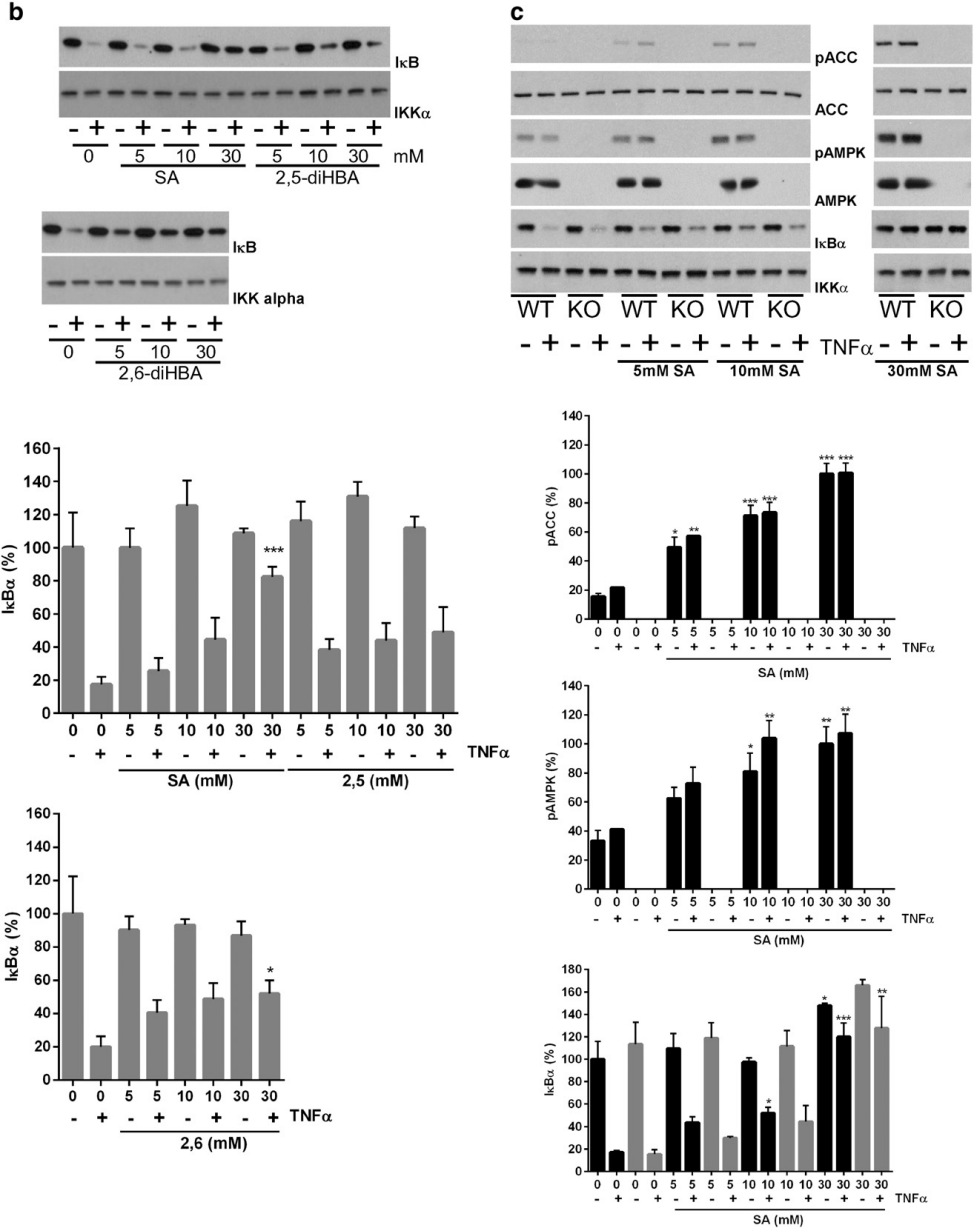
*Dose–response of SA and its analogues on signalling in wild type and AMPK double knockout primary hepatocytes*. (*a*, *b*) Primary mouse hepatocytes were treated in the presence or absence of 5–30 mM SA and the analogues shown for 3 h prior to treatment with 10 ng/ml TNFα (final 15 min). Signalling responses were measured using antibodies described in Fig. 1, Fig. 2. Densitometry of blots was carried out as described in Materials and methods. Treatments significantly different from the appropriate control column (+/− TNFα) are shown; ^⁎⁎⁎^p < .001, ^⁎⁎^p < 0.01, ^⁎^p < .05, n = 4–5. (*c*) Cells were treated with varying doses of SA as in (a), except that hepatocytes extracted from wild-type (WT, black bars) mice were compared side by side with those extracted from liver-specific AMPK double knockout (KO, grey bars) animals. Treatments significantly different from the appropriate control column (+/− TNFα, KO/wild-type) are shown, n = 3.

The results from HT-29 cells prompted us to study whether effects on inflammatory signalling may be observed in primary hepatocytes, which provide an excellent model to study glucose production in the laboratory. In signalling experiments, we found that the compounds exhibited similar effects to those in cell lines, with SA being the only compound giving robust AMPK pathway activation (Fig. 3a) and as in HT29 cells, both SA and 2,6-DHBA blocked TNF-induced IκB degradation at 30 mM (Fig. 3b). Since 2,6-DHBA is a very poor AMPK activator compared with SA (Figs. 1a,3a), this raised the possibility that this series of drugs might regulate IκB degradation independently of AMPK. Using hepatocytes extracted from liver-specific double knockout AMPK mice [22], we confirmed that the effect of SA on TNF-induced IκB degradation did not require AMPK (Fig. 3c).

### 3.4 Effect of salicylate and its analogues on glucose production and glucose 6-phosphatase (G6Pase) promoter expression

**Fig. 4:**
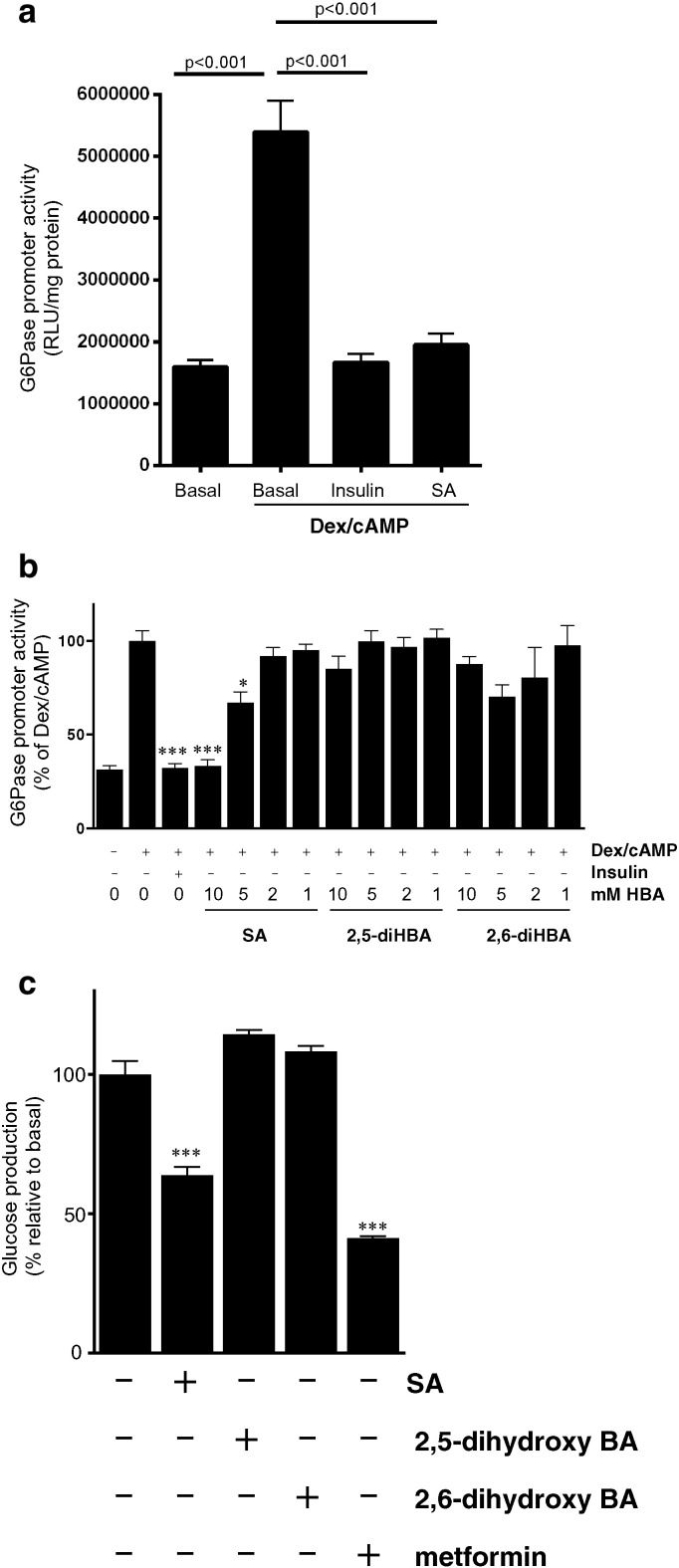
*Effect of salicylate and analogues on glucose 6-phosphatase promoter activity and glucose production.* (*a*) LLHG cells stably expressing the G6Pase promoter with a luciferase reporter were serum starved for 6 h and subsequent overnight treatment without or with a dose–response of 10 mM SA or insulin (10 nM) followed by lysis and measurement of luciferase as described in Materials and methods. (*b*) Dose–response of G6Pase promoter to SA, 2,5-DHBA and 2,6-DHBA. Drug treatments significantly different from Dex/cAMP are shown, ***p < .001, *p < .05 (*c*) Glucose production in response to 10 mM of each agent (except 2 mM metformin) was measured as described in the methods. Treatments significantly different from untreated cells are shown.

Earlier studies had suggested that SA acts by inhibition of gluconeogenesis, [17] and consistent with this, we observed an effect of 10 mM SA on promoter activity of the key gluconeogenic regulatory enzyme G6Pase (Fig. 4a). In dose–response experiments and similar to effects of metformin [1], we found that SA reduced expression of G6Pase promoter activity but in contrast, the other agents in the panel were unable to match this effect (Fig. 4b). Thus, G6Pase promoter activity, like AMPK activity, is unique to SA and correlates well with anti-hyperglycaemic properties in the drug series. Measuring effects on glucose, used at 10 mM, SA reduced glucose production, but in contrast, there was no response to 2,5-DHBA and 2,6-DHBA (Fig. 4c).

### 3.5 Effect of individual signalling pathways on glucose production

**Fig. 5:**
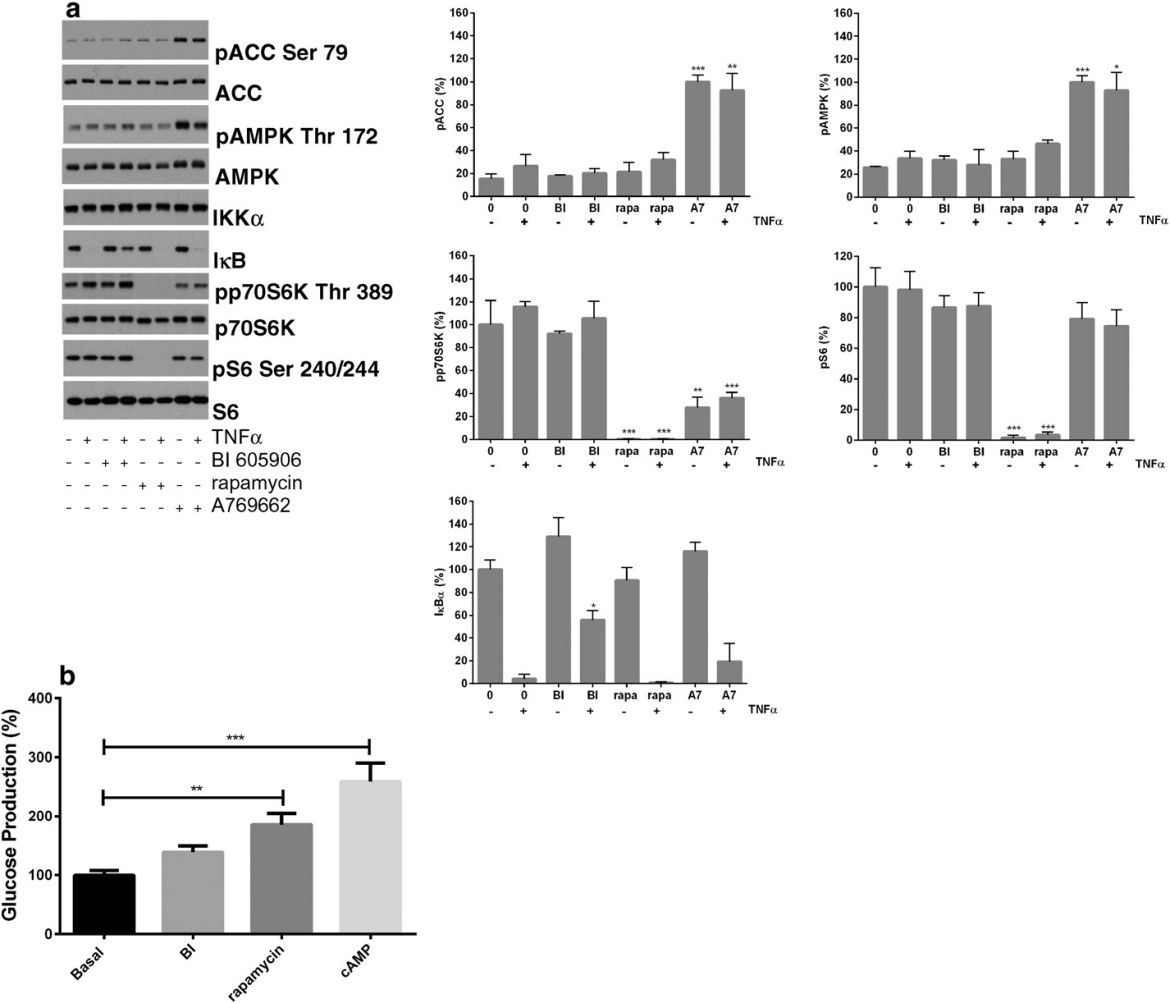
*Effect of specific inhibitors of mTOR and NF-κB on signalling and hepatocyte glucose production*. (*a*) Primary hepatocytes were pre-treated as shown with or without 10 μM BI605906 or 150 nM rapamycin prior to treatment with 10 ng/ml TNF-α (final 15 min), prior to lysis and immunoblotting using antibodies described earlier. Densitometry of blots was carried out as described in Materials and methods. Treatments significantly different from control cells (+/- TNF-α) cells are shown, n = 3. (*b*) Glucose production in response to each agent was measured as described in Materials and methods. ^⁎⁎⁎^p < .001 of treated columns with respect to no treatment.

Previous results suggested that AMPK activation is not sufficient to inhibit glucose production in hepatocytes [22]. In order to determine whether mTOR pathway or NF-κB inhibition might mediate effects of SA on glucose production, we incubated hepatocytes in the presence of a pharmacological agents to inhibit these pathways. We used rapamycin as a very potent inhibitor of mTOR [25], [26] and BI605906 to inhibit IKKβ and hence NF-κB signalling [41]. In these experiments, despite producing the expected effects on their respective pathways (we included A769662 as a positive control to demonstrate activation of AMPK signalling [42]) (Fig. 5a), neither of the agents were able to reduce hepatocyte glucose production (Fig. 5b).

### 3.6 Effect of selected agents on mitochondrial responses

**Fig. 6:**
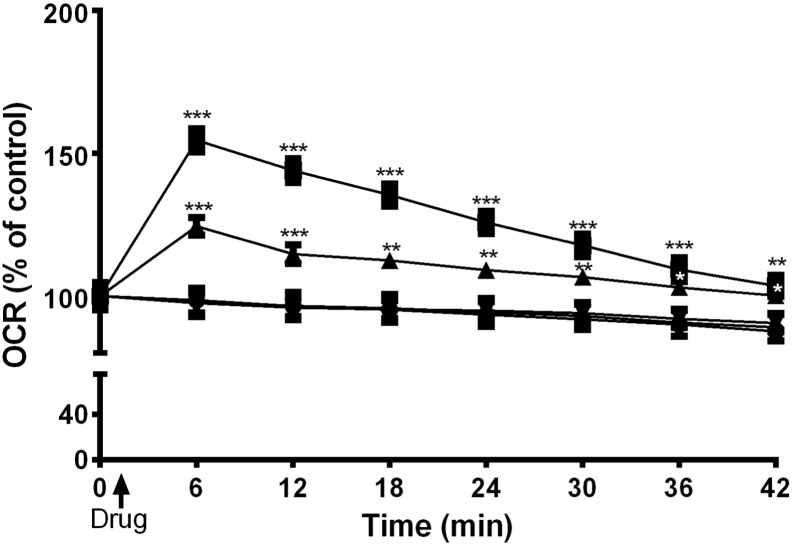
*Effect of salicylate, 2,5-DHBA, 2,6-DHBA and 2,4-dinitrophenol on mitochondrial respiration.* H4IIE cells were incubated in serum-free medium for 2 h followed by a further 1 h degas. Using a Seahorse analyser (Seahorse Bioscience), oxygen consumption rate (OCR) was measured. After the first reading, 2 mM salicylate (▲), 2 mM 2,5-DHBA (▼), 2 mM 2,6-DHBA (♦), or 100 μM 2,4-dinitrophenol (■) was added. Untreated samples are also shown (●). Data were normalised to untreated samples at zero minutes. Data are from 5 to 10 wells in duplicate. ^⁎⁎⁎^p < .001, ^⁎⁎^p < .01, ^⁎^p < .05 of treated time point with respect to no treatment at the same time point.

Earlier work comparing SA and the compound 2,4-dinitrophenol (DNP) attributed anti-hyperglycaemic effects to mitochondrial uncoupling induced by these agents [43], [44]. DNP has previously been shown to suppress glucose production, including experiments in hepatocytes [45] and in liver perfusion experiments [44]. To test whether mitochondrial effects might contribute to the difference between SA and 2,5- or 2,6-DHBA, we compared them in H4IIE cells, side by side with DNP in Seahorse experiments. Our experiments found that in H4IIE cells SA, but not 2,5- or 2,6-DHBA rapidly increased oxygen consumption within minutes of application, on a time course very similar to DNP (Fig. 6). The magnitude and potency of the two drugs were different however, as 2 mM SA produced about half the increase in oxygen consumption of cells treated with 100 μM DNP.

## 4 Discussion

Beginning our studies, we confirmed that salicylic acid (SA) activated AMPK and found that it repressed mTOR pathway signalling. Previous work has shown that these pathways are regulated in common by clinically used type 2 diabetes (T2D) agents metformin [5], [33], [34] and thiazolidinediones [1], [8]. In addition, SA inhibited TNFα-dependent NF-κB signalling as has been reported previously [20]. This raised the possibility that SA might exert some of its effects through AMPK-dependent regulation of inflammatory signalling [46]; however, in liver cells with both catalytic subunits of AMPK knocked out, the effect of SA on TNFα-dependent IκB degradation remained, indicating that effects of SA on NF-κB do not require AMPK.

In further work on a subgroup of the structures (results for this subgroup summarised in Table 1), we found that SA alone suppressed G6Pase promoter activity and hepatic glucose production. The concentrations we used to study these last two readouts (10 mM) were higher than concentrations of SA typically achieved in the general circulation, but earlier work has reported effects of aspirin, which is rapidly metabolised to salicylate, on glucose production with plasma concentrations in the range of 1.8–2.5 mM [47]. Similar results have been obtained in perfused liver, with effects of SA observed at 2 mM [17]. Due to pharmacokinetic considerations, the notion of gluconeogenesis as an important anti-hyperglycaemic target of SA might also help to account for the high doses of drug required for anti-hyperglycaemic effects. Radiolabelled salicylate and aspirin both distribute mainly to the stomach mucosa at low doses, but at higher doses, each of the main gluconeogenic tissues including liver, renal cortex, and the gastrointestinal tract are among the most strongly labelled tissues [48], [49], [50]. The side effects accompanying high doses of SA have greatly hindered adoption of this drug and related substances in T2D. Better targeting of gluconeogenic tissues may circumvent these difficulties.

Comparing signalling responses to SA and its analogues, we found that effects on AMPK phosphorylation corresponded most closely with effects on G6Pase promoter activity, hepatic glucose production, and published anti-hyperglycaemic properties of the drugs *in vivo*. Previous work has shown, however, that glucose production can be suppressed [22] and SA can mediate anti-hyperglycaemic effects [12] in AMPK-deficient mice. In addition to these earlier findings, we found that significant suppression of hepatocyte NF-κB signalling with salicylate required concentrations (10–30 mM) that are unlikely to be tolerably achieved *in vivo*. Furthermore, we found that inhibition of either mTOR or NF-κB was insufficient to suppress hepatic glucose production. Together, these results suggest that models of salicylate's anti-hyperglycaemic action based purely on signalling effects may be too simple and that other aspects need to be considered. Besides AMPK signalling, which needed at least 5–10 mM SA to be significantly activated in dose-response experiments, the only other action of hydroxybenzoic acids that we found correlated well with published anti-hyperglycaemic properties was mitochondrial uncoupling. SA but not its analogues was able to induce uncoupling acutely at 2 mM, a concentration which is consistent with ranges of plasma salicylate observed in humans following anti-hyperglycaemic doses of prodrugs salsalate or aspirin [47], [51]. Taken together with our data, this suggests that direct effects of SA on hepatic mitochondria are likely to contribute to anti-hyperglycaemic effects of SA, through action on hepatic gluconeogenic gene expression and hepatic glucose production. Mitochondrial inhibition has also previously been suggested as an anti-hyperglycaemic mechanism for biguanides [22] and thiazolidinediones [52]. The differences between SA and 2,5/2,6-DHBA we have observed might also account for previous findings that SA but not 2,5- nor 2,6-DHBA reduces liver glycogen stores [53]. In direct comparison, the SA effect on oxygen consumption was at a magnitude that was about half that of DNP, even though twenty times more SA was added than DNP. Further investigation of the reason(s) underlying this difference will be critical to illuminate the marked difference in the therapeutic window between these agents. It has long been recognised that SA is not only more efficacious but also much less toxic than DNP [43].

We do not exclude involvement of NF-κB, mTOR, or other signalling in anti-hyperglycaemic effects through other tissues and/or pathophysiological contexts *in vivo*. Protective effects of IKKβ knockout against insulin resistance, for example, are understood to be mediated mainly through effects that this has on alleviating systemic inflammation in obesity [54], [55]. To investigate this, it might be interesting to compare long-term effects of 2,6-DHBA and SA in diet-induced obese animals, as we found both drugs inhibit NF-κB signalling. 2,6-DHBA tends to reduce glucose tolerance, at least in short-term treatment [36], does not readily inhibit the mitochondria (our work and [13], [16]), and in our studies, it did not inhibit G6Pase promoter activity, nor did it reduce glucose output from hepatocytes; however, in the context of obesity and long-term drug treatment, beneficial effects of both drugs on inflammation may be exhibited, allowing comparison with pharmacology restricted to SA, such as the uncoupling effect that we have studied.

## 5 Conclusion

In this work, we have investigated responses to SA in hepatocytes. Comparison with a panel of SA analogues suggests that mitochondrial uncoupling and AMPK activation but not other signalling pathways correlate well with published anti-hyperglycaemic effects.

## Transparency Document

Transparency Document. Image 1

## Figures and Tables

**Table 1:** Comparison of effects of HBAs SA, 2,5-DHBA, and 2,6-DHBA.

Compound	Reduced glucose production (this study)	Activation of AMPK activity (this study and [12])	Uncoupling (this study)	Blockade of TNFα-induced NF-κB signalling in HT-29 cells (this study and [18])	> 50% inhibition of G6Pase promoter (this study)
SA	Yes	Yes	Yes	Yes	Yes
2,5-DHBA (gentisate)	No	No	No	No	No
2,6-DHBA (γ-resorcylate)	No	No	No	Yes	No
